# Outcomes of Anlotinib Maintenance Therapy in Patients With Advanced NSCLC in a Real-World Setting

**DOI:** 10.3389/fonc.2022.785865

**Published:** 2022-06-30

**Authors:** Jia Li, Baohui Han, Huaimin Liu

**Affiliations:** ^1^ Department of Integrated Chinese and Western Medicine, The Affiliated Cancer Hospital of Zhengzhou University & Henan Cancer Hospital, Zhengzhou, China; ^2^ Department of Respiratory Medicine, Shanghai Chest Hospital, Shanghai Jiao Tong University, Zhengzhou, China

**Keywords:** non-small cell lung cancer (NSCLC), maintenance therapy, effica cy, safety, anlotinib

## Abstract

**Background:**

Anlotinib is a novel tyrosine kinase inhibitor with promising anti-tumor activity in patients with advanced non-small cell lung cancer (NSCLC). The aim of this study was to evaluate the efficacy and safety of anlotinib maintenance therapy in patients with advanced NSCLC in real-world situation.

**Methods:**

We conducted a retrospective study on the patients with stage IIIb or IV NSCLC who visited our hospital between December 2016 and April 2020. They achieved an objective response or stable disease after first- or second- line treatment and then received switch maintenance therapy with anlotinib. Data were collected using automated data mining technology from electronic health records. Patients’ demographic and clinical characteristics, median progression-free survival (PFS), median overall survival (OS), and adverse events were described, and preliminary analysis of efficacy predictors was analyzed.

**Results:**

Thirty-two patients were included in this study (20 patients in first-line treatment and 12 patients in second-line treatment). Median anlotinib maintenance time was 9 days (Q1-Q3: 3-22). The median PFS was 11.5 months, with 11.5 months in first-line treatment and 8.9 months in second-line treatment. The median OS was 12.0 months, with 16.4 months in first-line treatment group and 8.9 months in second-line treatment group. Grade 2-3 treatment-related adverse events were reported in 18.76% patients. No life-threatening adverse events were observed.

**Conclusion:**

Our results suggested that anlotinib maintenance therapy might be a potentially safe and efficacious option for patients who had benefited from first- or second-line treatment. However, our data are limited representative due to the small sample size; the efficacy of anlotinib maintenance therapy warrants further evaluation.

## Introduction

Lung cancer is the most commonly diagnosed cancer worldwide and is one of the leading causes of cancer-related death, accounting for 20% of cancer-related deaths worldwide, with 1.77 million new deaths annually (1). Currently, there are approximately 0.733 million new cases and 0.61 million new deaths each year in China (1, 2). Recent data indicate that the incidence of lung cancer is dramatically rising with nearly half of new cases, 49.9%, diagnosed in the under-developed world (3). Non-small cell lung cancer (NSCLC) comprises 80% to 85% of lung cancers, with most patients diagnosed at locally advanced or advanced stages.

In recent years, the use of targeted therapies, including tyrosine kinase inhibitors (TKIs) (4) and immune checkpoint inhibitors (5, 6) has significantly prolonged overall survival (OS) of patients with advanced NSCLC. However, for most patients with advanced NSCLC, there is generally a brief period of disease control after response to first-line treatment, and patients will die of disease progression (7, 8). Consequently, it is needed to identify more effective and tolerable treatments to delay progression and improve survival in advanced NSCLC. Maintenance therapy, including continuation maintenance and switch maintenance, has emerged as a novel treatment strategy for advanced NSCLC, and has shown beneficial efficacy and safety in non-progressing NSCLC patients (9). At present, three drugs have been approved as maintenance monotherapy after platinum-based induction chemotherapy, including two targeted drugs and one cytotoxic drug: erlotinib, as switch maintenance for patients with any NSCLC histology; bevacizumab, as continuation maintenance after platinum-based and bevacizumab-containing triplet induction for patients with non-squamous NSCLC; and pemetrexed as switch or continuation maintenance for patients with non-squamous NSCLC.

Anlotinib is a novel oral small molecule TKI that targets angiogenesis pathways by inhibiting vascular endothelial growth fator receptor (VEGFR), platelet-derived growth factor receptor (PDGFR), fibroblast growth factor receptor (FGFR), and stem cell growth factor receptor (c-Kit). This drug also targets RET and other proteins, thereby inhibiting tumor proliferation (10, 11). It has demonstrated anti-tumor angiogenesis and tumor cell growth inhibition activity with good safety. The China Food and Drug Administration (CFDA) approved anlotinib for third-line-and-beyond treatment of advanced NSCLC based on the ALTER 0303 trial (12).

During the outbreak of COVID-19, we observed that some patients with advanced NSCLC appeared to benefit from anlotinib maintenance therapy after they had received and responded to the first- or second- line treatment. However, currently, there are no data regarding anlotinib switch maintenance therapy for advanced NSCLC patients in first- and second- line setting. Therefore, we conducted a retrospective study which described the real-world usage of anlotinib maintenance therapy for advanced NSCLC. The main objectives were: 1) to describe the demographic and clinical characteristics of patients receiving anlotinib maintenance therapy in Chinese patients with advanced NSCLC who achieved an objective response or stable disease (SD) after first- or second- line treatment in real world situations; 2) to investigate PFS and OS outcomes in this population; 3) to observe the safety profile and perform preliminary prognosis analysis.

## Methods

### Data Source and Study Population

This study was a retrospective multicenter analysis. Patients with advanced NSCLC who achieved an objective response or SD after first- or second- line chemotherapy-based treatment and then received switch maintenance therapy with anlotinib in our hospital from December 2016 to April 2020 were included in this study. The eligibility criteria included: diagnosis of NSCLC with pathological stage IIIb or IV; and anlotinib administered as monotherapy for patients who achieved an objective response or SD after first- or second- line treatment. The exclusion criteria included contraindication for anlotinib, concomitant with another primary cancer, and unavailable efficacy or follow-up data. The present study was approved by the ethics committee of our hospital (approval number: 2021-405-001). As this retrospective study did not harm the rights and health of patients, and protected their privacy and personal information, the ethics committee waived the requirement to obtain informed consent.

### Administration of Anlotinib

Anlotinib was orally administered at a dosage of 12 mg per day for 14 days and discontinued for 7 days in every 3-week cycle. Patients continued anlotinib until disease progression. Dose modification of anlotinib (10 or 8 mg/day) was allowed.

### Study Variables and Outcomes

The electronic health records (EHR) of the eligible patients were extracted from a cooperative medical intelligence platform (LinkDoc inc., China). By taking advantage of computer programming language and automated data mining technology, the demographic and clinical characteristics of all the patients were collected and then were reviewed by two physicians as following. Baseline variables included gender, age, BMI, smoking status, disease stage at diagnosis, ECOG PS, sites of metastases and best response to first- or second- line therapy (complete response, partial response, stable disease). Tumor response was also retrospectively captured and defined as reduction in burden of disease over the course of first-line or second-line therapy. Response was assessed based on the treating physician’s assessment or interpretation of the subsequent radiographic scans as described in the medical records. Treatment variables included first- or second- line treatment regimens received (e.g. drugs, duration of induction treatment) for all patients and anlotinib maintenance regimen (e.g. dosage, duration of maintenance treatment). Outcome variables were collected including included progression status, date of death, and adverse events (AE).

Primary outcome was progression-free survival (PFS) which was defined as the time from the first dosing of first-line or second-line treatment to tumor progression or patient death from any cause, whichever was earlier. Disease progression was retrospectively captured by using medical case notes in which the treating clinician documented that there had been growth or worsening of the disease. Secondary outcomes included overall survival (OS), 1-year PFS, median number of anlotinib maintenance therapy, anlotinib dosage reduction, safety and hospital stay. OS was defined as the time from the first dosing of first-line or second-line treatment to patient death from any cause. Hospital stay was defined as total inpatient stays during anlotinib administration. Death data was collected from the hospital’s EHR source and related follow-up data sources. If an event of interest was not observed, patients were censored at last follow-up. AEs were collected from the hospital’s EHR source and graded according to the Common Terminology Criteria for Adverse Events version 4.0. The data cutoff was December 7th 2020.

### Statistical Analysis

Statistical analysis was processed using SAS version 9.4. Results are presented in a descriptive fashion with continuous variables presented as mean ± SD, or median/interquartile range (IQR), and categorical variables as number/percentage. Descriptive statistics were used to describe the patient population. Survival curves were created using the Kaplan–Meier method. Cox regression estimated the statistically significant factors in univariate analysis and multivariate analysis. Two-sided test was performed with P <0.05 defined as statistical significance. Missing data were not imputed unless otherwise specified.

## Results

### Patient Characteristics

In total, 32 patients met the inclusion criteria and were enrolled into this study; of these, 27 (84.4%) patients were males, 25 (78.1%) aged >60 years, and 22 (68.75%) had a smoking history. Eight patients had the ECOG record, with ECOG PS ranging from 0 to 2. Other clinical characteristics of the patients are listed in **Table 1**. The genetic aberration status of EGFR, ALK (anaplastic lymphoma kinase) and MET (c-Mesenchymal-epithelial transition factor) was available in 19 patients. Among them 2 patients had tumors harboring EGFR aberration, 1 patient harboring EGFR and ALK aberrations, and 1 patient harboring EGFR and MET aberrations.

**Table 1:** Baseline characteristics of patients.

	First-line therapy (N = 20)	Second-line therapy (N = 12)	Total (N = 32)
**Sex, n (%)**			
Male	16 (80.0)	11 (91.7)	27 (84.4)
**Age (year)**			
Mean (SD)	64.5 (8.8)	64.5 (8.9)	64.5 (8.7)
**BMI (kg/m^2^)**			
Total (missing)	14 (6)	11 (1)	25 (7)
Mean (SD)	22.2 (2.1)	23.2 (2.4)	22.7 (2.3)
**Smoking, n (%)**			
Current smoker	6 (30.0)	4 (33.3)	10 (31.3)
Former smoker	9 (45.0)	3 (25.0)	12 (37.5)
Never	5 (25.0)	5 (41.7)	10 (31.3)
**Differentiation, n (%)**			
Total (missing)	7 (13)	7 (5)	14 (18)
High	3 (42.9)	3 (42.9)	6 (42.9)
Moderately high	0 (0.0)	1 (14.3)	1 (7.1)
Moderate	1 (14.3)	1 (14.3)	2 (14.3)
Low	3 (42.9)	2 (28.6)	5 (35.7)
**Pathologic type, n (%)**			
Adenocarcinoma	11 (57.9)	7 (58.3)	18 (58.1)
Squamous carcinoma	4 (21.1)	5 (41.7)	9 (29.0)
Others	4 (21.1)	0 (0.0)	4 (12.9)
**TNM stage, n (%)**			
IIIB	0 (0.0)	2 (16.7)	2 (6.3)
IV	20 (100.0)	10 (83.3)	30 (93.8)
**Metastasis, n (%)**			
Yes	15 (75.0)	7 (58.3)	22 (68.9)
**Metastatic sites, n (%)**			
Lung	3 (15.0)	1 (8.3)	4 (12.5)
Liver	3 (15.0)	1 (8.3)	4 (12.5)
Brain	4 (20.0)	0 (0.0)	4 (12.5)
Bone	5 (25.0)	4 (33.3)	9 (28.1)
Kidney	3 (15.0)	1 (8.3)	4 (12.5)
Pleural	2 (10.0)	0 (0.0)	2 (6.3)
Abdomen	2 (10.0)	0 (0.0)	2 (6.3)
Pelvic	1 (5.0)	0 (0.0)	1 (3.1)
**ECOG PS, n (%)**			
Total (missing)	3 (17)	5 (7)	8 (24)
0	0 (0.0)	1 (20.0)	1 (12.5)
1	1 (33.3)	4 (80.0)	5 (62.5)
2	2 (66.7)	0 (0.0)	2 (25.0)

Among the 32 patients, 20 (62.5%) patients received first line treatment and 12 (37.5%) received second line treatment before anlotinib maintenance therapy (**Supplementary Table 1**). Twenty-one patients (21/32, 65.6%) received chemotherapy alone, with 14 patients for first-line therapy and 7 for second-line therapy. The chemotherapy regimens included docetaxel monotherapy (1/32, 3.1%), gemcitabine monotherapy (1/32, 3.1%), pemetrexed monotherapy (1/32, 3.1%), paclitaxel monotherapy (1/32, 3.1%), platinum + docetaxel (7/32, 21.9%), platinum + gemcitabine (2/32, 6.3%), platinum + pemetrexed (5/32, 15.6%), platinum + etoposide (2/32, 6.3%), and platinum + paclitaxel (1/32, 3.1%). Nine patients (9/32, 28.1%) received chemotherapy combined with targeted therapy, with 5 patients for first-line therapy and 4 for second-line therapy. The regimens included platinum + docetaxel platinum + bevacizumab (1/32, 3.1%), platinum + gemcitabine + recombinant human endostatin (1/32, 3.1%), platinum + pemetrexed + bevacizumab (2/32, 6.3%), platinum + pemetrexed + recombinant human endostatin (1/32, 3.1%), platinum + paclitaxel + bevacizumab (2/32, 6.3%), pemetrexed + icotinib (1/32, 3.1%), and pemetrexed + bevacizumab (1/32, 3.1%). Two patients (2/32, 6.3%) received chemotherapy combined with immunotherapy for first-line (platinum + paclitaxel + Nivolumab) and for second-line therapy (platinum + S-1 + fluorouracil + Nivolumab) respectively.

### Maintenance Therapy With Anlotinib

The median total anlotinib administration was 108mg (Q1-Q3: 32-264), with 21 patients (65.6%) receiving >50 mg. The median anlotinib maintenance time was 9 days (Q1-Q3: 3-22). The median dosage of anlotinib was 12mg (Q1-Q3: 10-12); dose reduction occurred in 3 patients (3/27, 11.11%) who initially received 12 mg anlotinib and then were adjusted to 10 mg. The cause of the dose reductions in these patients was unclear.

### Efficacy of Anlotinib Maintenance

**Figure 1:**
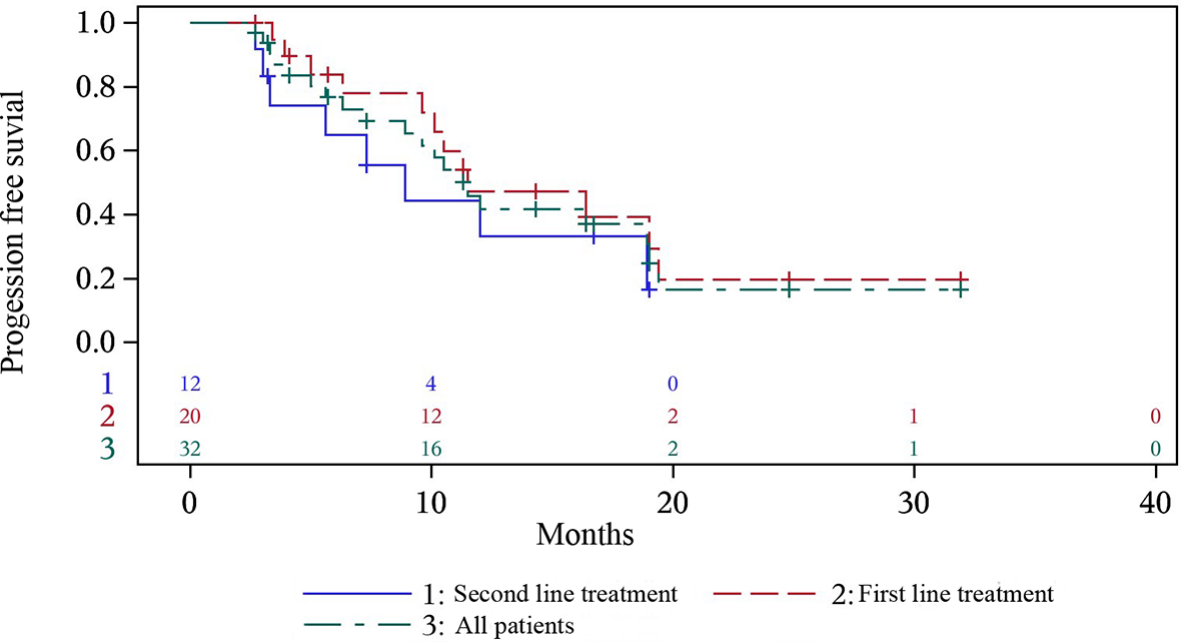
Progression-free survival of all the patients, and first- and second- line treatment group.

On the basis of a data cutoff date of December 7th 2020, 7 (21.9%) patients had disease progression after anlotinib maintenance with 6 patients in the first-line treatment group and 1 in the second-line treatment group, and 13 (40.6%) patients died before disease progression with 6 patients in the first-line treatment group and 7 patients in the second-line treatment group. The median (95% confidence interval [CI]) PFS was 11.5 months (7.3-19.0; **Figure 1**), with median PFS of 11.5 months (9.6, 19.4) in first-line treatment and 8.9 months (3.0, 18.9) in second-line treatment. 1-year PFS and 0.5-year PFS were 47.2% (22.7%, 68.3%) and 83.9% (57.9%, 94.5%) respectively in first-line treatment group. 1-year PFS and 0.5-year PFS were 33.3% (8.5%, 61.3%) and 64.8% (31.0%, 85.2%) respectively in second-line treatment group. Multivariate analysis indicated that former smoker (HR=4.352, P=0.0462) and distant metastasis (HR=17.761, P=0.0098) were independent risk factors for a shorter PFS (**Table 2**).

**Table 2:** Multivariate analysis for predictors of progression-free survival and overall survival.

Characteristic	Progression-free survival		Overall survival	
	HR (95%CI)	P value	HR (95%CI)	P value
Age ≥60 years	0.630 (0.135, 2.942)	0.5565	0.698 (0.143, 3.414)	0.6573
Former smoker	4.352 (1.025, 18.470)	**0.0462**	4.077 (0.906, 18.334)	0.0670
Current smoker	3.442 (0.501, 23.631)	0.2085	3.019 (0.442, 20.628)	0.2599
Squamous carcinoma	0.509 (0.095, 2.743)	0.4323	0.614 (0.123, 3.063)	0.5518
Others (non-adenocarcinoma)	0.912 (0.218, 3.817)	0.9001	0.812 (0.160, 4.117)	0.8014
Second-line treatment	1.859 (0.540, 6.396)	0.3255	3.101 (0.850, 11.306)	0.0864
Metastasis	17.761 (2.000, 157.748)	**0.0098**	18.469 (2.082, 163.808)	**0.0088**
Anlotinib administration >14 days	2.766 (0.738, 10.369)	0.1314	1.575 (0.388, 6.394)	0.5249

This means that P value is less than 0.05, which indicates statistically significant.

**Figure 2:**
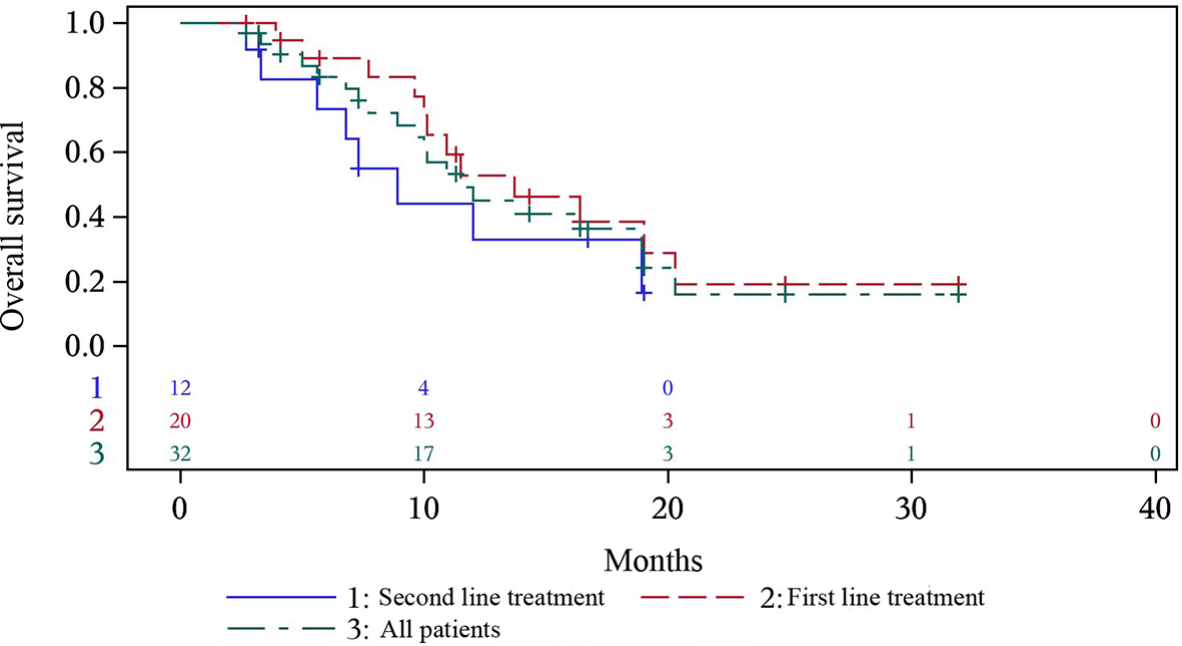
Overall survival of all the patients, and first- and second- line treatment group.

At the time of data cutoff, 17 (53.1%) patients died. The median (95% CI) OS was 12.0 months (8.9-19.0), with median OS of 16.4 months (10.1-NC) in first-line treatment group and 8.9 months (3.0-18.9) in second-line treatment group (**Figure 2**). 0.5-year OS rate and 1-year OS rate were 89.2% and 57.2% respectively in first-line treatment group and were 73.3% and 33.0% respectively in second-line treatment group. Multivariate analysis indicated that only patients with distant metastasis (HR=18.469, P=0.0088) had a shorter OS (**Table 2**).

### Safety Outcomes

Among the 32 patients, any treatment-related adverse events (TRAEs) were reported in 15 patients (46.88%), including hemoglobin decline (18.75%), creatinine increase (9.38%), hypoalbuminemia (6.25%), leukopenia (6.25%), and cholesterol increase (6.25%). Grade 2 TRAEs were reported in 5 (15.63%) patients and grade 3 TRAEs in 1 (3.13%) patient. No serious or life-threatening adverse events were reported.

## Discussion

At present, no clinical trials or real-world studies have investigated the efficacy and safety of maintenance therapy with anlotinib in NSCLC. To our best knowledge, this study is the first to assess the efficacy and safety of anlotinib maintenance for advanced NSCLC in first- and second- line setting. Our results suggested that anlotinib maintenance therapy might be a potentially safe and efficacious option for patients who achieved an objective response or SD after chemotherapy-based treatment for NSCLC in first- or second-line setting.

Multiple maintenance therapies for advanced NSCLC have shown potential benefits to non-progressing patients. Some trials on maintenance therapy randomized treatments only to patients not progressing after first-line chemotherapy, and subsequently measured survival and progression time from start of maintenance therapy (13–15); while many other trials randomized patients at onset of first-line therapy, and subsequently measured survival and progression time from start of first-line therapy, then through maintenance (16, 17). In this real-world study, we enrolled the non-progressing patients after receiving first- or second- line treatment and measured PFS and OS from the onset of first- or second- line therapy.

The outbreak of COVID-19 has been causing a lot of inconvenience for those advanced NSCLC patients who need to be hospitalized for treatment. During our clinical practice, we observed that those patients appeared to have benefited from anlotinib maintenance therapy which could be orally administered at home, thus it might allow postponed inpatient treatment. The present study showed a median PFS and OS of 11.5 and 12.0 months respectively in all patients, with 11.5 and 16.4 months in first-line treatment. The median PFS in the present study was a little longer than that (10.3 months) in a retrospective observational bevacizumab maintenance study (18) which only included the patients in first line setting while the present study also included 12 (37.5%) patients in the second line setting. Several recent observational studies which used bevacizumab as maintenance therapy after induction have shown an outstanding OS of 19-20 months (18–20). Similarly, the results from the maintenance analysis of the ARIES study support the hypothesis that maintenance bevacizumab therapy provides a potential benefit in effectiveness outcomes (median PFS and OS of 9.2 and 19.8 months respectively) after induction treatment (21). However, the median OS in first-line treatment group of this study was shorter (16.4 months *vs* 19-20 months), which might be partly attributed to the poor ECOG PS of the patients in this study as the previous report showed better survival in subjects with good ECOG PS as compared to those with poor ECOG PS (22). Similarly, maintenance chemotherapy with pemetrexed in the PARAMOUNT trial reported a PFS and OS of 6.9 and 16.9 months respectively (23, 24), while in a phase II study in NSCLC patients with an ECOG PS of 2, pemetrexed monotherapy resulted in median PFS and OS of 3.0 and 9.5 months, respectively (25). Unfortunately, in this study the ECOG PS record was only available in eight patients. Considering this limited data, we did not perform a sub-analysis or further discuss the influence of ECOG PS to the median PFS and OS in this study.

Additionally, key trials in the first-line setting have shown little or no survival benefit from first-line therapies with maintenance regimens in unselected populations (13–15). Nevertheless, survival benefits have been observed from some patients who were selected by particular biomarkers (26). In this study, multivariate analysis indicated that only patients with distant metastasis had a shorter PFS and a shorter OS. However, the sample is too limited to define the patient population who will benefit most from the anlotinib maintenance. For the future, we may need to involve more patients and collect more marker information, such as EGFR mutation status, to narrow the core patients who could derive benefit from maintenance therapy with anlotinib.

In this study, the AE profile during anlotinib maintenance was different from those in the clinical trials of anlotinib, which was probably due to the nature of real-world study and the small sample size (11). In this study, AEs were reported in 15 patients (46.88%), including one case (3.13%) of grade 3 AE; no serious AEs were observed.

There were some limitations to the present study. Firstly, this is a retrospective study with a small sample size, thus the data had inherent limitations. Secondly, some pathological subtypes included only four or five patients, and the number of metastatic foci was not available. Thirdly, the OS data requires longer follow-up to evaluate whether maintenance therapy with anlotinib would improve the overall survival. A larger observational study should be conducted to confirm the efficacy and toxicity of anlotinib maitenance for the patients with advanced NSCLC.

In conclusion, this study describes a group of patients with advanced NSCLC who had benefited from first- or second-line chemotherapy-based treatment followed by anlotinib maintenance. Our results showed that switch maintenance therapy with anlotinib might be a potentially safe and efficacious option for patients with locally advaced or metastatic NSCLC who had responded with at least SD to first- or second-line treatment. It may probably provide a new option for the maintenance treatment of advanced NSCLC. However, with limited data in this study, the definite efficacy of anlotinib switch maintenance warrants further evaluation.

## Data Availability Statement

The raw data supporting the conclusions of this article will be made available by the authors, without undue reservation.

## Ethics Statement

The studies involving human participants were reviewed and approved by Ethics committee of Henan Cancer Hospital. Written informed consent for participation was not required for this study in accordance with the national legislation and the institutional requirements.

## Author Contributions

JL: Study design, data collection, data analysis and manuscript draft. BH: Study design, data collection, data analysis and manuscript revision. HL: Study design, data collection, data analysis and manuscript revision. All authors contributed to the article and approved the submitted version.

## Funding

This study was supported by Joint Fund Project (NSFC-Henan United Fund. U2004105); General Project of Henan Provincial Natural Science Foundation (No.202300410450); Key R&D and promotion projects in Henan Province (212102310342); Henan Province Medical Science and Technology Research Project (SBGJ202103032); Henan Province Traditional Chinese Medicine Research Project (2022ZY1224).

## Conflict of Interest

The authors declare that the research was conducted in the absence of any commercial or financial relationships that could be construed as a potential conflict of interest.

## Publisher’s Note

All claims expressed in this article are solely those of the authors and do not necessarily represent those of their affiliated organizations, or those of the publisher, the editors and the reviewers. Any product that may be evaluated in this article, or claim that may be made by its manufacturer, is not guaranteed or endorsed by the publisher.
