# Supplementary material for: Outcomes of Anlotinib Maintenance Therapy in Patients With Advanced NSCLC in a Real-World Setting
Source: Front Oncol. 2022 Jun 30;12:785865. doi: 10.3389/fonc.2022.785865 (PMC9282872; doi:10.3389/fonc.2022.785865)
Supplement: Supplementary file 1 [file Table_1.docx]

Supplementary table-1. Treatment cycles before anlotinib maintenance therapy

| **Treatment cycles** | **First-line therapy (N=20)** | **Second-line therapy (N=12)** | **Total  (N=32)** |
| --- | --- | --- | --- |
| Number (missing) | 20 (0%) | 12 (0%) | 32 (0%) |
| 1 cycle | 5 (25.00%) | 0 (0.00%) | 5 (15.63%) |
| 2 cycles | 3 (15.00%) | 0 (0.00%) | 3 (9.38%) |
| 3 cycles | 4 (20.00%) | 3 (25.00%) | 7 (21.88%) |
| 4 cycles | 2 (10.00%) | 0 (0.00%) | 2 (6.25%) |
| 5 cycles | 3 (15.00%) | 3 (25.00%) | 6 (18.75%) |
| 6 cycles | 2 (10.00%) | 1 (8.33%) | 3 (9.38%) |
| 7 cycles | 0 (0.00%) | 2 (16.67%) | 2 (6.25%) |
| 8 cycles | 1 (5.00%) | 1 (8.33%) | 2 (6.25%) |
| 10 cycles | 0 (0.00%) | 1 (8.33%) | 1 (3.13%) |
| ≥10 cycles | 0 (0.00%) | 1 (8.33%) | 1 (3.13%) |

Data are presented as number (percentage).
